# Study on effects and relevant mechanisms of Mudan granules on renal fibrosis in streptozotocin-induced diabetes rats

**DOI:** 10.1080/0886022X.2024.2310733

**Published:** 2024-02-15

**Authors:** Mushuang Qi, Xiangka Hu, Wanjun Zhu, Ying Ren, Chunmei Dai

**Affiliations:** a Jinzhou Medical University, Jinzhou, Liaoning, China; b Institute of Materia Medica, Jinzhou Medical University, Jinzhou, Liaoning, China

**Keywords:** Mudan granules, diabetic nephropathy, fibrosis, TGF-β1/smad

## Abstract

**Aims:**

The effects and relevant mechanisms of Mudan granules in the renal fibrosis of diabetic rats were explored through *in vivo* experiments, which provided a scientific basis for expanding their clinical indications.

**Methods:**

Male SD rats were given a single intraperitoneal injection of STZ (65 mg/kg) to induce diabetes rat models. After treatment with Mudan granules, the general condition of rats was recorded. Blood glucose, blood lipids, and renal function-related indicators were detected, renal tissue morphological changes and fibrosis-related indicators were observed, and the expression of pathway-related proteins were examined.

**Results:**

The general condition of diabetes rats was improved after the treatment of Mudan granules, the 24-h urinary protein and urinary albumin to creatinine ratio were reduced, and the renal function and lipid results were modified. The tissue damage to the rat kidney has been repaired. Expression of TGF-β1/Smad-related pathway proteins was suppressed in kidney tissues, and the fibrosis factor CO-IV, FN, and LN were reduced in serum.

**Conclusion:**

Mudan granules may inhibit of TGF-β1/Smad pathway, inhibit the production of ECM, reduce the levels of fibrosis factors CO-IV, FN, and LN, to have a protective effect on kidney in diabetes rats.

## 1. Introduction

Diabetes mellitus (DM) can cause a variety of microvascular complications, of which diabetes nephropathy (DN) is one of the common complications [1]. Diabetes nephropathy is the main cause of end-stage renal nephropathy, and more and more patients with diabetes nephropathy develop end-stage renal disease [2]. During the progression of disease, continuous hyperglycemia will lead to abnormal glomerular blood flow stability, abnormal vascular permeability, and extracellular matrix (ECM) deposition, eventually leading to glomerulosclerosis and renal fibrosis [3]. Therefore, the inhibition of glomerulosclerosis and renal fibrosis is an effective strategy to prevent DN from developing into end-stage nephropathy. Under high blood sugar conditions [4], in order to repair kidney damage caused by adverse stimuli, the body needs to activate the ECM repair mechanism of the kidney. The ECM mainly includes fibronectin, collagen, laminin, and proteoglycans. The continuous deposition of ECM ultimately leads to renal fibrosis [5]. A large number of studies have been shown that the activation of transforming growth factor(TGF) and its downstream signal cascade plays an important role in diabetes nephropathy. Among them, Smad signaling pathway is the main pathway related to renal fibrosis [6]. Confirmed, TGF-β1 binds to its receptor, then activates its downstream signaling molecules Smad2 and Smad3. After activation, it binds to Smad4 to form a complex that enters the nucleus, regulating target gene transcription, mRNA expression, and protein generation as key components of ECM. Smad7 plays a negative feedback role in this signaling pathway [7, 8]. Thus, TGF-β1 is considered as a potential biomarker for renal fibrosis and inhibiting TGF-β1/Smad2/3 pathway may represent an effective therapy for DN associated with progressive real fibrosis [9].

Mudan granules are traditional Chinese patent medicines and simple preparations, which has been widely used in clinical practice. Mudan granules are composed of Huangqi (*Astragalus*), Yanhusuo (*Corydalis*), Sanqi (*Panaxnotoginseng*), Chishao (*Radix paeoniae rubra*), Danshen (*Salvia miltiorrhiza*), Chuanxiong (*Ligusticum chuanxiong hort*), Honghua (*Carthamus tinctorius*), Sumu (*Logwood*), Jixueteng (*spatholobi Caulis*) [10]. Many studies have been shown that it has anti-inflammatory, anti-oxidative stress, improving vascular endothelial function and other effects, and can improve microcirculation. It was first used in patients with diabetes peripheral neuropathy, and had a good effect. It also has certain therapeutic effect on other complications of diabetes [11].

The purpose of this study was to explore the protective effect of Mudan granules on kidney of diabetes rats and its relevant mechanisms and to provide a basis for expanding its clinical indications.

## 2. Materials and methods

### 2.1. Drug

Mudan granules were provided by Liaoning Oda Pharmaceutical Co., Ltd (batch number: 10211019, Liaoning, China). Valsartan capsules were sourced from Hainan Aomei Pharmaceutical Co., Ltd (batch number: 01211106, Hainan, China).

### 2.2. Animal experiments

Male Sprague–Dawley rats with weights ranging from 180 to 220 g were purchased from Liaoning Changsheng Biotechnology Co., Ltd. with license number SCXK (Liao) 2020-0001. All rats were fed in a 12 h light/dark circulation chamber at a temperature of 23 ± 1 °C and were free to get food and water.

Forty SD male rats were randomly divided into a normal control group (*n* = 5) and a modeling group (*n* = 35). Based on previous experience and pre-experiments, the Type I Diabetes mellitus (T1DM) model was induced by a single intraperitoneal injection of strepotozotocin (STZ, 65 mg/kg, in 0.1 mol/L sodium citrate buffer, pH 4.4; Sigma, United States) in SD rats, and the normal control group was injected with an equal volume of citrate buffer at the same time. After 72 h of STZ injection, animals with random blood glucose levels ≥16.7 mmol/L were considered successful in modeling [12, 13].

Subsequently, diabetic rats were randomly divided into 5 groups (*n* = 5 for group): diabetic model group, STZ + Valsartan capsules (Valsartan, 8.23 mg/kg), STZ + low dose Mudan granules (MD 1.08 g/kg), STZ + medium dose Mudan granules (MD 2.16 g/kg), STZ + high dose Mudan granules (MD 4.32 g/kg). The dosage of valsartan and Mudan granules is calculated based on the daily dose commonly used in humans through conversion. According to the third edition of ‘Methodology of Pharmacological Research on Traditional Chinese Medicine’ edited by Chen Qi, the dose relationship between adults and rats was converted to obtain the dosage for rats. The clinical dosage of Mudan granules was selected as the medium dose, with the low dose being half of the medium dose and the high dose being twice the medium dose. Deionized water or drug was treated by gavage administration once a day (10 mL/kg body weight) for 12 consecutive weeks. Body weight were measured at every week in each group. The food intake, water consumption, fasting blood glucose (FBG) and random blood glucose (RBG) levels of the rats were monitored at 0, 2, 4, 6, 8, 10, 12 weeks. Urine samples were collected for 24-h after 12 weeks. After intraperitoneal injection of 20% urethane anesthesia in rats, the abdominal aorta was found under anesthesia and blood was collected using a blood collection needle and negative pressure tube. The blood samples were centrifuged at 3500 rpm for 10 min at 4 °C The supernatant was collected and stored in *a* − 80 °C refrigerator. Rats were euthanized under anesthesia and their kidneys were quickly removed. One part was fixed in 10% formalin, and the other part was immediately stored at −80 °C until further analysis. All animal experiments were approved by the Ethics Committee of Jinzhou Medical University (No. 2022030502).

### 2.3 24-h urine test

Urine of rats was collected using metabolism cages, in which rats had free access to water. The 24-h urine was centrifuged at 4 °C at 2000 rpm for 5 min, and store the supernatant at −80 degrees Celsius. Detection of urinary protein and creatinine levels in urine using a reagent kit (NO: C035-2-1; NO: C011-2-1; Nanjing Jiancheng Institute of Biological Engineering, Nanjing, China). Then, calculated the ratio of urine protein to urine creatinine.

### 2.4. Serum sample index detection

The contents of the plasma lipid profile of triacylglycerol (TG; A110-1-1), total cholesterol (TC; A111-1-1), low-density lipoprotein cholesterol (LDL-C; A113-1-1), high-density lipoprotein cholesterol (HDL-C; A112-1-1), serum creatinine (Scr; C011-2-1) and blood urea nitrogen (BUN; C013-2-1) in the serum were detected by Nanjing Jiechen Kits (Nanjing Jiancheng Institute of Biological Engineering, Nanjing, China). The levels of fibronectin (FN; YX-061400R), laminin (LN; YX-121400R) and collagen type IV (CO-IV; YX-031512R) were measured using appropriate ELISA kits (all from R&D Systems).

### 2.5. Histopathological tests

The kidneys of rats were embedded in paraffin after dehydration and then the tissue was sliced for pathological staining by hematoxylin-eosin staining (HE staining), periodic acid-Schiff (PAS) and Masson staining methods. The change of renal structure was observed under light microscope.

### 2.6. Western blotting

The kidney tissue (100 mg) was homogenized on ice in 1 mL RIPA (Beyotime Biotechnology, Shanghai, China) containing 1% PMSF and 2% phosphatase inhibitor, stewed and then centrifuged at 12000 rpm for 10 min at 4 °C. The supernatants were collected. The protein lysate concentration was measured for protein concentration. After using the BCA protein concentration determination kit (Beijing Dingguo Changsheng biotech CO. LTD, Beijing, China) to determine the protein concentration, proteins (20 μg) were resolved by 10% SDS-polyacrylamide electrophoresis gel electrophoresis (SDS-PAGE) which was followed by a transfer of proteins from gels to the nitrocellulose filter membranes. Next, the membranes were incubated with primary antibodies overnight at 4 °C, including TGF-β1 (A2124, 1:1000), Smad2 (A7699, 1:1000), p-Smad2 (18338, 1:1000), Smad3 (A19115, 1:1000), p-Smad3 (9520, 1:1000), Smad7(A16396,1:1200) and β-actin (AC026, 1:1000). The above-mentioned primary antibodies were obtained from ABclonal (ABclonal Technology Co., Ltd., Wuhan, China) except p-Smad2 and p-Smad3 which were from Cell Signaling Technology (CST, Danvers, MA, United States). The membrane was incubated with the appropriate secondary antibodies coupled to horseradish peroxidase and the blots were developed with the chemiluminescence reagent using an enhanced chemiluminescence kit (Biosharp, Hefei, China).

### 2.7. Statistical analysis

The data were presented as the mean ± standard deviation. Statistical analysis was carried out using SPSS 26.0. The differences among multiple groups were evaluated by one-way analysis of variances. Pairwise comparisons that satisfy homogeneity of variance use LSD method, while those that do not satisfy homogeneity of variance use Games–Howell method. *p* < 0.05 was considered as statistically significant, while *p* < 0.01 was considered as a significant statistical difference.

## 3. Results

### 3.1. The effect of Mudan granules on the general condition and blood glucose in the diabetic rats

**Figure 1.**
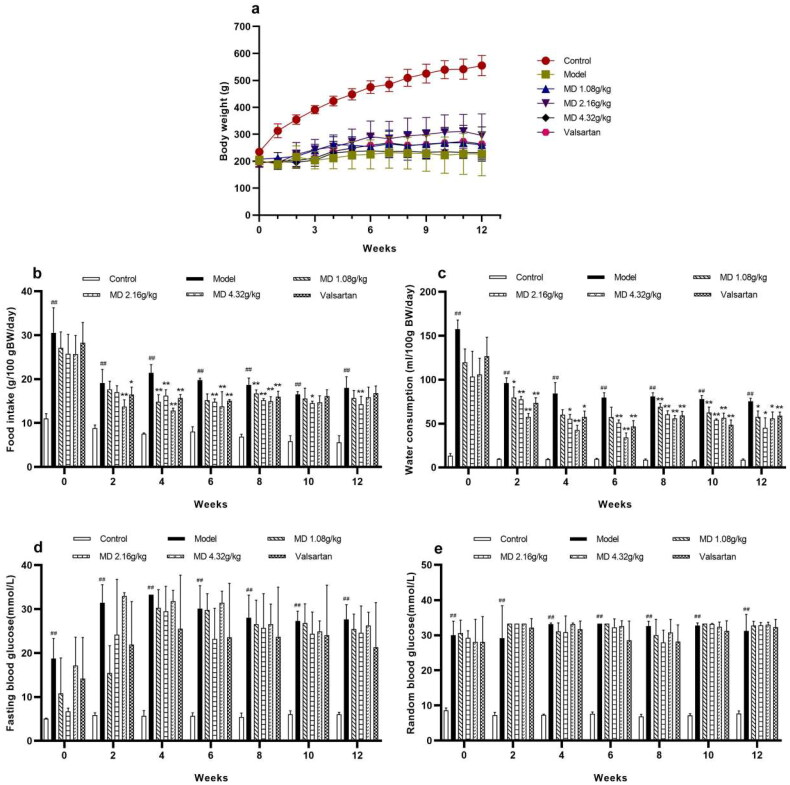
Effect of Mudan granules on weight, food intake, water consumption and blood glucose in diabetic rats. a: Body weight of rats. b: Food consumption. c: Water consumption. d: Fasting blood glucose. e: Random blood glucose. All data were expressed as mean ± SD, n = 5. Compared with the control group, ^#^*P* < 0.05, ^##^*P* < 0.01. Compared with the diabetic model group, *P < 0.05, **P < 0.01.

Compared with the control group, the weight of rats in the model group was remarkably reduced, the food intake and water consumption of rats were statistically evaluated in the model group. Compared with the model group, the food intake and water consumption were statistically sensitively depressed in the valsartan and each dose group of Mudan granules (Figure 1(a–c)).

The fasting blood glucose (FBG) and random blood glucose (RBG) levels of the rats were monitored at 0, 2, 4, 6, 8, 10, 12 weeks after 12 h of fasting. FBG and RGB were significantly increased in the model group and maintained at a higher level throughout the experimental period (Figure 1(d,e)).

### 3.2. The effect of Mudan granules on the function of the kidney in diabetic rats

**Figure 2.**
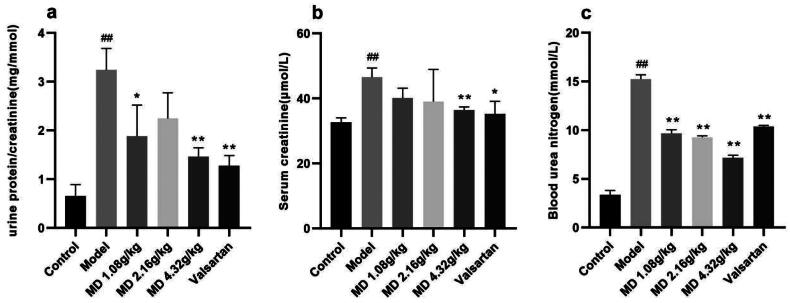
Effect of Mudan granules on the function of the kidney in diabetic rats. a: UACR. b: Serum creatinine. c: Blood urea nitrogen. All data were expressed as mean ± SD, n = 5. Compared with the control group, ^#^*P* < 0.05, ^##^*P* < 0.01. Compared with the diabetic model group, *P < 0.05, **P < 0.01.

Compared with the control group, rats in the model group showed significantly increased in 24-h urinary protein and showed significant decreased in creatinine. The level of 24-h urinary protein in the high dose group of Mudan granules was significantly decreased in comparison with the model group and the level of creatinine was significantly increased in comparison with the model group (Table 1). Compared with the control group, rats in the model group showed significantly increased in UACR (urinary albumin to creatinine ratio). The level of UACR in the low-dose and high-dose groups of Mudan granules was significantly decreased in comparison with the model group (Figure 2(a)).

**Table 1.** The effect of Mudan granules on 24-h urinary protein and creatinine in diabetic rats.

	24-h urinary protein(mg/L)	Creatinine(mmol/L)
Control	231.75 ± 86.58	351.17 ± 27.58
Model	678.23 ± 83.20^##^	209.80 ± 11.95^##^
MD 1.08 g/kg	443.48 ± 138.35	237.34 ± 20.72
MD 2.16 g/kg	497.96 ± 114.42	222.29 ± 24.97
MD 4.32 g/kg	364.74 ± 23.91*	252.33 ± 36.45**
Valsartan	329.43 ± 13.14**	262.97 ± 44.76**

All data were expressed as mean ± SD, *n* = 5. Compared with the control group, ^#^*p* < 0.05, ^##^*p* < 0.01. Compared with the diabetic model group, *p < 0.05, **p < 0.01.

Compared with the control group, rats in the model group showed significant differences in Scr and BUN. Compared with the model group, the Scr was significantly improved in the high dose group of Mudan granules and valsartan treatment group, and BUN was significantly improved in all dosing groups (Figure 2(b,c)).

### 3.3. The effect of Mudan granules on blood lipids in diabetic rats

**Figure 3.**
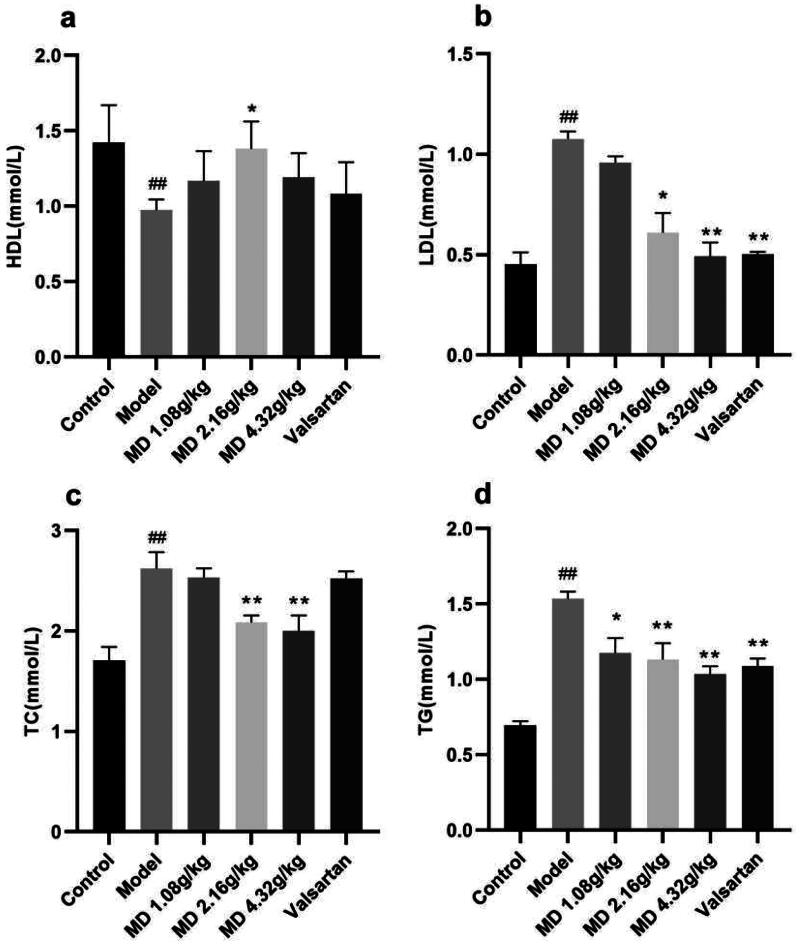
Effect of Mudan granules on blood lipids in diabetic rats. a: High-density lipoprotein cholesterol. b: Low-density lipoprotein cholesterol. c: Total cholesterol. d: Triglyceride. All data were expressed as mean ± SD, n = 5. Compared with the control group, ^#^*P* < 0.05, ^##^*P* < 0.01. Compared with the diabetic model group, *P < 0.05, **P < 0.01.

Compared with the control group, the serum levels of TG, TC and LDL-C in rats of the diabetes model group were significantly increased and HDL-C levels were significantly decreased. Compared with the model group, the serum levels of TG and LDL-C in rats of the valsartan group were decreased to different degrees. The levels of HDL-C in the serum of rats in the middle dose group of Mudan granules was increased, and the levels of TG, TC and LDL-C in the serum of rats in the middle and high dose groups of Mudan granules were decreased to some extent (Figure 3).

### 3.4. The effect of Mudan granules on morphology of the kidney in diabetic rats

**Figure 4.**
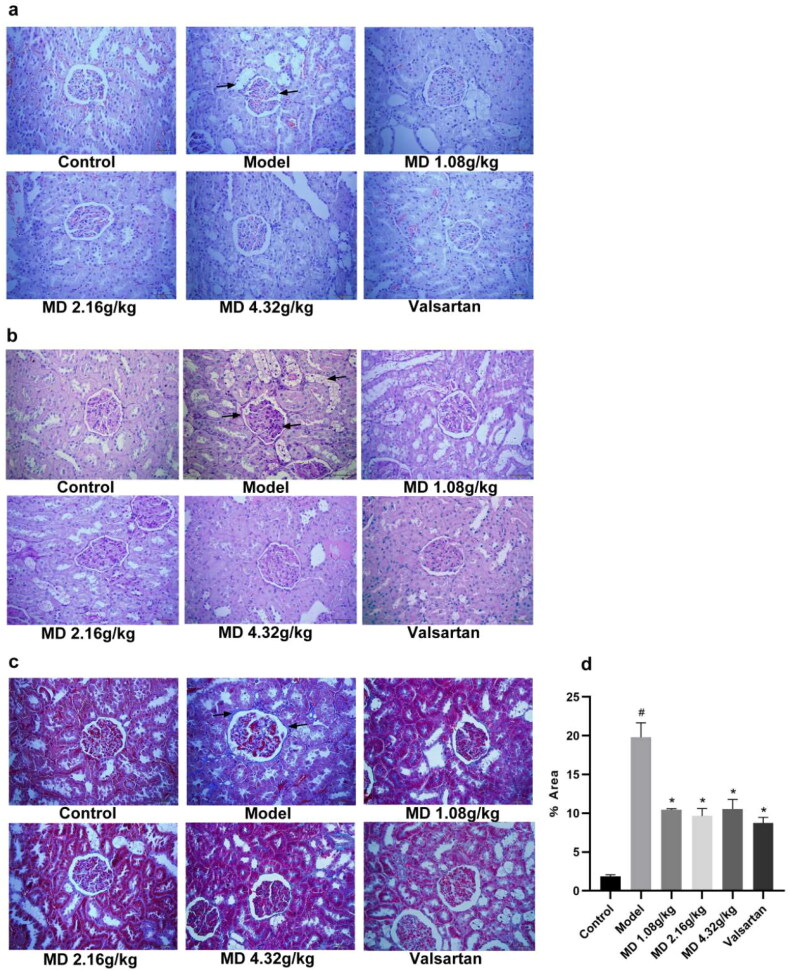
Effect of Mudan granules on the histomorphology of the kidney in diabetic rats (×400). a: HE staining. The black arrows point to fat vacuoles and irregular glomerular morphology. b: PAS staining. The black arrows point to fat vacuoles and glycogen deposition. c: Masson staining. The black arrows point to collagen fibers. d: Renal fibrosis area (%) in each group. All data were expressed as mean ± SD, n = 3. Compared with the control group, ^#^*P* < 0.05, ^##^*P* < 0.01. Compared with the diabetic model group, *P < 0.05, **P < 0.01.

Based on HE staining, PAS staining and Masson staining, no pathological changes were observed in the kidney tissue of the control group, while the glomerular morphology was irregulared, the mesangial matrix was increased, the glomerular basement membrane was thickened, the arrangement of renal tubules was disordered, the fat vacuoles was appearance in the model group. Compared with the model group, the degree of lesion was reduced to some extent in the each treatment groups (Figure 4).

Meanwhile, quantitative analysis of Masson staining results revealed that fibrosis levels were significantly higher in the model group than in the control group, and after treatment, fibrosis was improved in the Mudan granules treatment groups and valsartan treatment group (Figure 4d).

### 3.5. The effect of Mudan granules on serum fibrosis factor levels in diabetic rats

**Figure 5.**
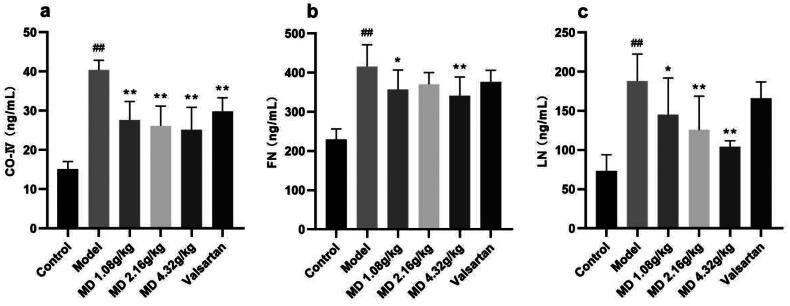
Effect of Mudan granules on serum fibrosis factor levels in diabetic rats. a: Collagen type IV. b: Fibronectin. c: Laminin. All data were expressed as mean ± SD, n = 5. Compared with the control group, ^#^*P* < 0.05, ^##^*P* < 0.01. Compared with the diabetic model group, *P < 0.05, **P < 0.01.

When compared with the control group, the serum CO-IV, FN and LN levels of rats in the diabetic model group were visibly elevated. Compared with the diabetic model group, the serum CO-IV and LN levels of rats in the three dose groups of Mudan granules were moderately declined to some extent, the serum FN levels of rats in the low and high dose groups of Mudan granules had decreased (Figure 5).

### 3.6. Effect of Mudan granules on the expression of TGF-β1/Smad2/3 pathway proteins in kidney tissues of diabetic rats

**Figure 6.**
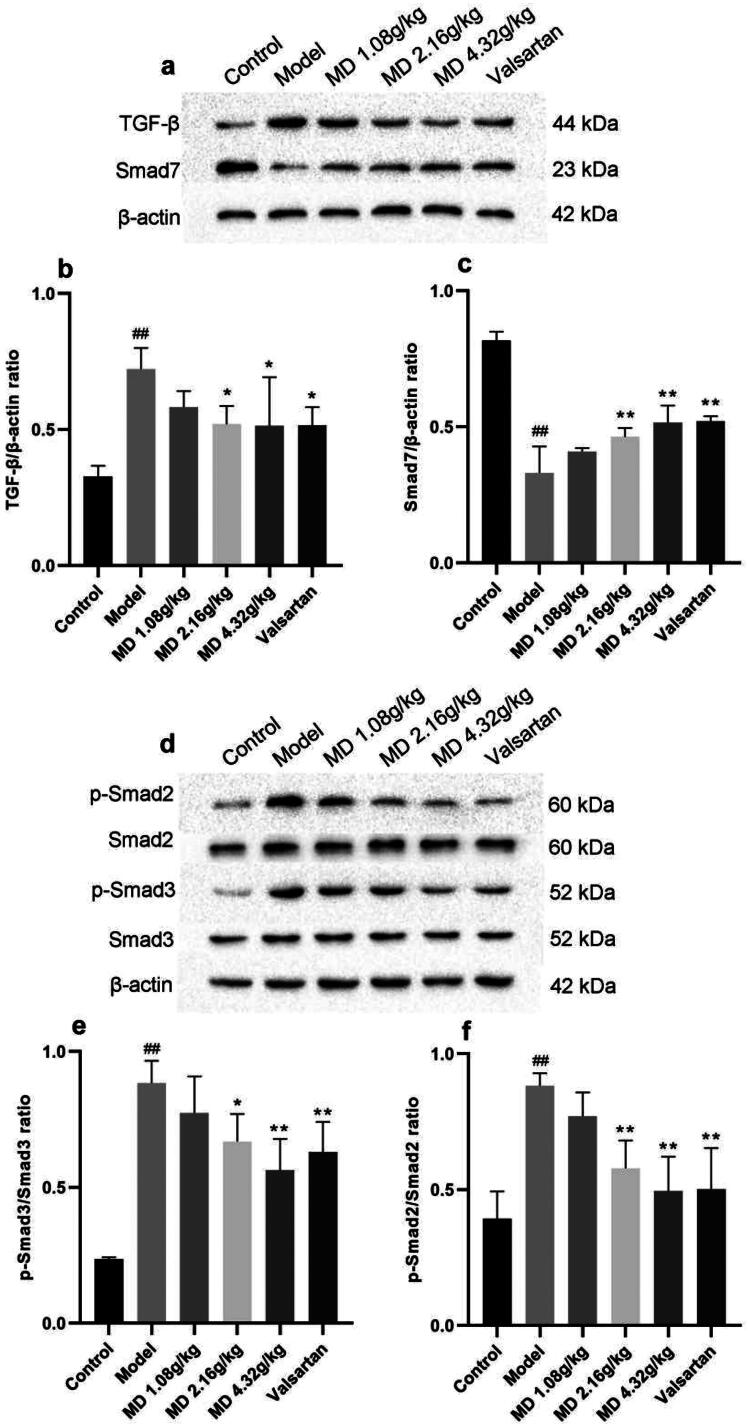
Effect of Mudan granules on the expression of TGF-β/Smad2/3 pathway proteins in kidney tissues of diabetic rats. a: TGF-β and Smad7 protein expression in kidney tissues of rats. b: TGF-β/β-actin ratio. c: Smad7/β-actin ratio. d: Smad2, Smad3 and phosphorylation protein expression in kidney tissues of rats. e: Smad2/p-Smad2 ratio. f: Smad3/p-Smad3 ratio. All data were expressed as mean ± SD, n = 3. Compared with the control group, ^#^*P* < 0.05, ^##^*P* < 0.01. Compared with the diabetic model group, *P < 0.05, **P < 0.01.

Compared with the control group, renal TGF-β1, p-Smad2, p-Smad3 protein expression was elevated hugely and Smad7 expression was declined in the model group rats, suggesting activation of TGF-β/Smad2/3 signaling pathway in the kidney. Compared with the model group, the middle and high dose groups of Mudan granules and valsartan group were able to reduce the protein expression of TGF-β1, p-Smad2, p-Smad3 and elevate Smad7 protein expression in the kidney of rats (Figure 6).

## 4. Discussion

It is efficient, simple and stable to establish an animal model of diabetes with streptozotocin (STZ) [14]. Valsartan capsules belong to the angiotensin II receptor antagonist (ARB) class of drugs. They can dilate the entering and exiting small arteries, reduce the three high states of high pressure, high perfusion, and high filtration in the glomerulus, reduce proteinuria, slow down glomerulosclerosis, and have a protective effect on the kidneys. In addition, there are nonblood pressure dependent mechanisms, such as inhibiting apoptosis of renal podocytes, inhibiting matrix deposition of mesangial cells caused by high glucose, and improving renal fibrosis [15–17].In this study, the blood glucose, food intake, water consumption, and urine volume were significantly increased, meanwhile the body weight of model rats was lower than normal group. Renal tissue section staining showed renal structural and pathological hallmarks of DN. These alterations were considered as evidence for establishing a model of renal damage in diabetes. This study revealed that Mudan granules could improve symptoms of T1DM, such as weight loss, polydipsia, and polyphagia. In our study, it is noteworthy that Mudan granules exerted no hypoglycemic action on rats, which indicated that Mudan granules potentially ameliorated DN without affecting the blood glucose level.

The classical presentation and evolution of DN is characterized by impaired glomerular filtration membrane barrier function in patients with diabetic nephropathy leads to increased excretion of albumin in urine. Then the renal function gradually decreased, serum creatinine (SCr) and blood urea nitrogen (BUN) increased [1, 2]. Renal pathological changes are the gold standard for diagnosis of diabetes nephropathy [18]. The pathological characteristics of diabetic nephropathy included the thickening of the glomerular basement membrane, diffuse mesangial expansion, the increase of inflammatory cells of renal tubule interstitium, renal interstitial fibrosis and glomerular sclerosis [19]. However, these are closely related to ECM deposition. Increased purple color in the model group after PAS staining indicated the deposition of glycogen in the kidneys, reflecting the glomerular basement membrane thickened and mesangial matrix expansion. Masson stained-kidneys showed an increase in blue color, indicating a large amount of collagen fibronectin deposition, which proved the interstitial fibrosis in the kidneys [3]. Our study showed that Mudan granules reduced urinary albumin, ameliorated SCr and BUN, and alleviated the pathological renal lesion in rats, suggesting the protective role of Mudan granules in the renal fibrosis of diabetic rats [20]. It could be seen that each dose group of Mudan granules has a certain effect, and the high-dose group of Mudan granules has the best effect.

Studies have shown that there is intimate crosstalk between lipid metabolism and renal fibrosis. Moreover, improving abnormal lipid metabolism in the kidney would effectively prevent and treat renal fibrosis [21]. The loss of urine protein causes compensatory synthesis of lipoproteins in the liver, and excessive free fatty acids or lipoproteins can cause damage to renal epithelial cells, thereby promoting the progression of kidney disease [22]. In our research, serum TG, TC, and LDL-C levels were reduced, and HDL-C levels were elevated, indicating that Mudan granules could ameliorate lipid metabolism homeostasis in diabetic rats. Moreover, it could be seen that the high-dose group of Mudan granules could better improve lipid metabolism abnormalities.

The progressive accumulation of extracellular matrix (ECM) components led to kidney fibrosis. Evidence indicates that collagen, FN, and LN increase significantly in diabetes-induced renal fibrosis [3, 5, 23]. Our study found that Mudan granules treatment during the last 12 weeks significantly inhibited T1DM-induced collagen, FN, and LN expression. TGF-β1 is a multifunctional cytokine that has been identified as a critical regulator of ECM protein synthesis in DN [14]. The studies had confirmed that TGF-β could stimulate mesangial cells, interstitial fibroblasts, and tubular epithelial cells to become ECM secreting fibroblasts through activation and transformation of myofibroblasts [24, 25]. Treatment factors such as high sugar and glycosylation induced downregulation of matrix metalloproteinases (MMPs) expression by TGF-β mediation, thereby inhibiting ECM degradation [26]. Therefore, the disruption of matrix synthesis and decomposition disrupts the physiological balance of ECM. Under physiological conditions, the healing of kidney injury occurs through TGF-β1/Smad signaling pathway mediating repair activities such as ECM generation and cell renewal; however, during the DN process, when kidney cells receive sustained stimulation and undergo excessive repair, pathological features of renal fibrosis such as cell proliferation and excessive matrix deposition occur [27].

Numerous studies have focused on inhibition of TGF-β1 and its downstream target genes for treatment of DN, and smad-dependent signaling pathway played a critical role in pathogenesis of DN. The major downstream mediators of TGF-β1 were Smad2 and Smad3. It has been reported the important role of Smad3 in renal fibrosis. It has been demonstrated that Smad3 mainly mediated renal fibrosis, and deletion of Smad3 reduced fibrogenesis [28, 29]. The important findings demonstrated that conditional deletion of Smad2 could significantly attenuate renal fibrosis [30]. Besides, Smad2 deletion promoted fibrosis through enhanced TGF-β/Smad3 signaling, and increased autoinduction of TGF-β1 [31]. Smad7, as a negative feedback regulator of TGF-β1/Smad signaling, is responsible for halting the recruitment and phosphorylation of Smad2 and Smad3 [32, 33]. After the activation of TGF-β/Smad pathway, Smad2 and Smad3 were overactivated, and Smad7 was downregulated [34]. Here, we found that Mudan granules treatment significantly inhibited TGF-β/Smad signaling to prevent the accumulation of ECM. Among them, the high-dose group was particularly remarkable.

In conclusion, Mudan granules could ameliorate the disorders of lipid metabolism, decreased urine protein, ameliorate renal function, reduce the degree of renal histopathological damage and fibrosis, retarded accumulation of ECM in rats. Its mechanism of action may be with the inhibition of TGF-β/Smad signaling pathway and inhibiting the expression of extracellular matrix components, which in turn suppresses the production of CO-IV, LN and FN, thus achieving an deferment of renal fibrosis in diabetic rats. This study suggests that Mudan granule may be a potential traditional Chinese patent medicines to prevent renal fibrosis in diabetes. However, the etiology and pathogenesis of renal fibrosis are complex, and the therapeutic and preventive effects of Mudan granules on diabetic rats still need to be further explored. This project has provided scientific support for the therapeutic application of Mudan granules in renal fibrosis of diabetes through basic research in the laboratory animals.

## Authorship contribution statement

All authors contributed to the study conception and design. Material preparation, data collection and analysis were performed by [Mushuang Qi], [Xiangka Hu], [Wanjun Zhu] and [Ying Ren]. Supervision and project administration were performed by [Chunmei Dai]. The first draft of the manuscript was written by [Mushuang Qi] and all authors commented on previous versions of the manuscript. All authors read and approved the final manuscript.

## Ethics approval

This study was performed in line with the principles of the Declaration of Helsinki. Approval was granted by the Ethics Committee of Jinzhou Medical University (No 2022030502).

## References

[1] Kushwaha K, Kabra U, Dubey R, et al. Diabetic nephropathy: pathogenesis to cure. Curr Drug Targets. 2022;23(15):1–10. doi: 10.2174/1389450123666220820110801. 35993461

[2] Samsu N. Diabetic nephropathy: challenges in pathogenesis, diagnosis, and treatment. Biomed Res Int. 2021;2021:1497449. doi: 10.1155/2021/1497449. 34307650 PMC8285185

[3] Pan Y, Zhang Y, Li J, et al. A proteoglycan isolated from Ganoderma lucidum attenuates diabetic kidney disease by inhibiting oxidative stress-induced renal fibrosis both in vitro and in vivo. J Ethnopharmacol. 2023;310:116405. doi: 10.1016/j.jep.2023.116405. 36966849

[4] Zhu D, Zhang L, Cheng L, et al. Pancreatic kininogenase ameliorates renal fibrosis in streptozotocin induced-diabetic nephropathy rat. Kidney Blood Press Res. 2016;41(1):9–17. doi: 10.1159/000368542. 26751697

[5] Zheng Z, Ma T, Lian X, et al. Clopidogrel reduces fibronectin accumulation and improves diabetes-induced renal fibrosis. Int J Biol Sci. 2019;15(1):239–252. doi: 10.7150/ijbs.29063. 30662363 PMC6329922

[6] Zhang ZH, Li MH, Liu D, et al. Rhubarb protect against tubulointerstitial fibrosis by inhibiting TGF-beta/smad pathway and improving abnormal metabolome in chronic kidney disease. Front Pharmacol. 2018;9:1029. doi: 10.3389/fphar.2018.01029. 30271345 PMC6146043

[7] Ma TT, Meng XM. TGF-beta/smad and renal fibrosis. Adv Exp Med Biol. 2019;1165:347–364. doi: 10.1007/978-981-13-8871-2_16. 31399973

[8] Zhao T, Sun S, Zhang H, et al. Therapeutic effects of tangshen formula on diabetic nephropathy in rats. PLoS One. 2016;11(1):e147693. doi: 10.1371/journal.pone.0147693. PMC4726711 26807792

[9] Zhang X, Zhao L, Xiang S, et al. Yishen tongluo formula alleviates diabetic kidney disease through regulating Sirt6/TGF-beta1/Smad2/3 pathway and promoting degradation of TGF-beta1. J Ethnopharmacol. 2023;307:116243. doi: 10.1016/j.jep.2023.116243. 36791927

[10] Zhang Y, Jin D, Duan Y, et al. Efficacy of mudan granule (combined with methylcobalamin) on type 2 diabetic peripheral neuropathy: study protocol for a Double-Blind, randomized, Placebo-Controlled, Parallel-Arm, Multi-Center trial. Front Pharmacol. 2021;12:676503. doi: 10.3389/fphar.2021.676503. 34093204 PMC8173202

[11] Xu J, Wang M, Yu S. Experimental and clinical research evidence of mudan granules (tangmoning) in the treatment of diabetes and multiple complications. Chinese Archives of Traditional Chinese Medicine. 2018;36(02):384–387.

[12] Zhang X, Zhang L, Chen Z, et al. Exogenous spermine attenuates diabetic kidney injury in rats by inhibiting AMPK/mTOR signaling pathway. Int J Mol Med. 2021;47(3):1–11. doi: 10.3892/ijmm.2021.4860. PMC7895520 33537831

[13] Liu Y, Zheng JY, Wei ZT, et al. Therapeutic effect and mechanism of combination therapy with ursolic acid and insulin on diabetic nephropathy in a type I diabetic rat model. Front Pharmacol. 2022;13:969207. doi: 10.3389/fphar.2022.969207. 36249783 PMC9561261

[14] Qi SS, Zheng HX, Jiang H, et al. Protective effects of chromium picolinate against diabetic-induced renal dysfunction and renal fibrosis in streptozotocin-induced diabetic rats. Biomolecules. 2020;10(3):398. doi: 10.3390/biom10030398. 32143429 PMC7175215

[15] Gao F, Yao M, Cao Y, et al. Valsartan ameliorates podocyte loss in diabetic mice through the notch pathway. Int J Mol Med. 2016;37(5):1328–1336. doi: 10.3892/ijmm.2016.2525. 26985716

[16] Jiao B, Wang YS, Cheng YN, et al. Valsartan attenuated oxidative stress, decreased MCP-1 and TGF-beta1 expression in glomerular mesangial and epithelial cells induced by high-glucose levels. Biosci Trends. 2011;5(4):173–181. doi: 10.5582/bst.2011.v5.4.173. 21914953

[17] Tang L, Yi R, Yang B, et al. Valsartan inhibited HIF-1alpha pathway and attenuated renal interstitial fibrosis in streptozotocin-diabetic rats. Diabetes Res Clin Pract. 2012;97(1):125–131. doi: 10.1016/j.diabres.2012.01.037. 22377232

[18] Oh SW, Kim S, Na KY, et al. Clinical implications of pathologic diagnosis and classification for diabetic nephropathy. Diabetes Res Clin Pract. 2012;97(3):418–424. doi: 10.1016/j.diabres.2012.03.016. 22521535

[19] Ricciardi CA, Gnudi L. Kidney disease in diabetes: from mechanisms to clinical presentation and treatment strategies. Metabolism. 2021;124:154890. doi: 10.1016/j.metabol.2021.154890. 34560098

[20] Zheng ZC, Zhu W, Lei L, et al. Wogonin ameliorates renal inflammation and fibrosis by inhibiting NF-kappaB and TGF-beta1/Smad3 signaling pathways in diabetic nephropathy. Drug Des Devel Ther. 2020;14:4135–4148. doi: 10.2147/DDDT.S274256. PMC7549498 33116403

[21] Dorotea D, Koya D, Ha H. Recent insights into SREBP as a direct mediator of kidney fibrosis via lipid-Independent pathways. Front Pharmacol. 2020;11:265. doi: 10.3389/fphar.2020.00265. 32256356 PMC7092724

[22] Sun J, Chen X, Liu T, et al. Berberine protects against Palmitate-Induced apoptosis in tubular epithelial cells by promoting fatty acid oxidation. Med Sci Monit. 2018;24:1484–1492. doi: 10.12659/msm.908927. 29528039 PMC5859669

[23] Kolset SO, Reinholt FP, Jenssen T. Diabetic nephropathy and extracellular matrix. J Histochem Cytochem. 2012;60(12):976–986. doi: 10.1369/0022155412465073. 23103723 PMC3527883

[24] Sureshbabu A, Muhsin SA, Choi ME. TGF-beta signaling in the kidney: profibrotic and protective effects. Am J Physiol Renal Physiol. 2016;310(7):F596–F606. doi: 10.1152/ajprenal.00365.2015. 26739888 PMC4824143

[25] Loboda A, Sobczak M, Jozkowicz A, et al. TGF-beta1/smads and miR-21 in renal fibrosis and inflammation. Mediators Inflamm. 2016;2016:8319283. doi: 10.1155/2016/8319283. 27610006 PMC5005604

[26] Chang AS, Hathaway CK, Smithies O, et al. Transforming growth factor-beta1 and diabetic nephropathy. Am J Physiol Renal Physiol. 2016;310(8):F689–F696. doi: 10.1152/ajprenal.00502.2015. 26719364 PMC4835922

[27] Gosmanov AR, Wall BM, Gosmanova EO. Diagnosis and treatment of diabetic kidney disease. Am J Med Sci. 2014;347(5):406–413. doi: 10.1097/MAJ.0000000000000185. 24553399

[28] Chen L, Yang T, Lu DW, et al. Central role of dysregulation of TGF-beta/smad in CKD progression and potential targets of its treatment. Biomed Pharmacother. 2018;101:670–681. doi: 10.1016/j.biopha.2018.02.090. 29518614

[29] Wang L, Wang HL, Liu TT, et al. TGF-Beta as a master regulator of diabetic nephropathy. Int J Mol Sci. 2021;22(15):1–18. 10.3390/ijms22157881 PMC8345981 34360646

[30] Nomura M, Li E. Smad2 role in mesoderm formation, left-right patterning and craniofacial development. Nature. 1998;393(6687):786–790. doi: 10.1038/31693. 9655392

[31] Hu HH, Chen DQ, Wang YN, et al. New insights into TGF-beta/smad signaling in tissue fibrosis. Chem Biol Interact. 2018;292:76–83. doi: 10.1016/j.cbi.2018.07.008. 30017632

[32] Heldin CH, Moustakas A. Role of smads in TGFbeta signaling. Cell Tissue Res. 2012;347(1):21–36. doi: 10.1007/s00441-011-1190-x. 21643690

[33] Yan X, Liao H, Cheng M, et al. Smad7 protein interacts with receptor-regulated smads (R-Smads) to inhibit transforming growth factor-beta (TGF-beta)/smad signaling. J Biol Chem. 2016;291(1):382–392. doi: 10.1074/jbc.M115.694281. 26555259 PMC4697173

[34] Liu S, Zhao J, Tian WS, et al. Estrogen deficiency aggravates fluorine ion-induced renal fibrosis via the TGF-beta1/smad signaling pathway in rats. Toxicol Lett. 2022;362:26–37. doi: 10.1016/j.toxlet.2022.04.005. 35504524

